# The complete mitochondrial genome of *Ips calligraphus* (Germar 1824) (Coleoptera: Curculionidae: Scolytinae)

**DOI:** 10.1080/23802359.2021.1958079

**Published:** 2021-07-27

**Authors:** Miao-Feng Xu, Rui Meng, Ke-Juan Shui, Bo Cai, Wei Lin

**Affiliations:** a Technical Center, Gongbei Customs District, Zhuhai, P. R. China; b China Post-Entry Quarantine Station for Tropical Plant, Haikou Customs District, Haikou, P. R. China

**Keywords:** *Ips calligraphus*, mitochondrial genome, Coleoptera

## Abstract

The complete mitogenome of *Ips calligraphus* was sequenced, the length was 19,144 bp which consists of 13 protein-coding genes, 22 transfer RNA genes, two ribosomal RNA genes, and a major non-coding AT-rich region (GenBank accession no. MW589547). All of 13 protein-coding genes (PCGs) started with ATN. 12 PCGs used the typical stop codon ‘TAA,’ while ATP8 terminated with stop codon ‘TAG.’ Phylogenetic analyses were performed using mitochondrial PCGs for the *I. calligraphus* and other 18 species within the Scolytinae. The *I. calligraphus* was clustered together with the other two *Ips* species in tribe Ipini which were closely related to Xyleborini and Dryocoetini.

*Ips calligraphus* (Germar 1824) is mainly a secondary pest of conifer forests and feeds mostly on *Pinus* spp. *Ips calligraphus* was native in North and Central America and the Caribbean Islands (Wood and Bright 1992). It was introduced to Benguet, Philippines in 1987 (Zamora 1987) and found in Guangdong, China recently.

Adult individuals of *I. calligraphus* were collected from Fenghuang mountain in Zhuhai Guangdong, China (22.29 N 113.52 E) on 31 October 2019. A specimen was deposited at −20 °C in the plant quarantine laboratory of the Technical Center of Gongbei Customs District, P. R. China (http://gongbei.customs.gov.cn/, Wei Lin, lw0525@163.com) under the voucher number: IC11.

The complete mitochondrial genome of *Ips calligraphus* (GenBank: MW589547) was sequenced using Illumina Novaseq 6000 platform and de novo assembly was conducted by MitoZ (Meng et al. 2019). The *I. calligraphus* mitochondrial genome is a closed circular molecule of 19,144 bp in length. The genome contains 13 Protein-coding genes (PCGs), 22 transfer RNA genes (tRNAs), two ribosomal RNA genes (rrnL and rrnS), and a non-coding AT-rich region. The complete mitochondrial nucleotide composition was biased to AT 75.7%, and the total base composition was 39.9% A, 35.8% T, 10.1% G, 14.2% C.

The total length of protein-coding genes was 10,716 bp. All PCGs start with the conventional initiation codons (ATN). Twelve PCGs shared the same complete stop codon (TAA), while the ATP8 gene used TAG as the stop codon. Individual tRNAs of *I. calligraphus* ranged from 63 bp (trnC) to 72 bp (trnE) in length. The large ribosomal gene (rrnL) was 1367 bp in length, which is located between trnL1 and trnV. The small ribosomal gene (rrnS) was 819 bp in length and positioned between trnV and the AT-rich region. The non-coding AT-rich region located in rrnS and trnI corresponding to the control region, with A + T contents, are 79.9%. Furthermore, there was an 1158 bp ‘supernumerary’ non-coding region between trnI and trnQ like some other species in Curculionidae.

Phylogenetic analysis of *I. calligraphus* with 18 other Scolytinae species was conducted based on nucleotide sequence data of 13 PCGs (Figure 1). The analysis was performed with Bayesian inference under GTR + F + I + G4 model in Phylosuite (Zhang et al. 2020). The phylogenetic tree showed that the relationship within Scolytinae was similar to the previous study (Vega and Hofstetter 2015). The *I. calligraphus* was clustered together with the other two *Ips* DeGeer, 1775 species in tribe Ipini which were closely related to Xyleborini and Dryocoetini.

**Figure 1.**
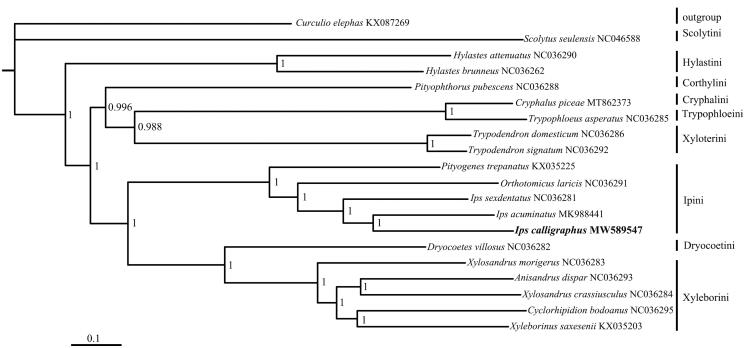
Phylogenetic tree showing the relationship between *Ips calligraphus* and other Scolytinae species based on Bayesian methods. Numbers on branches are Bayesian posterior probabilities.

## Data Availability

The genome sequence data that support the findings of this study are openly available in GenBank of NCBI at (https://www.ncbi.nlm.nih.gov/) under the accession no. MW589547. The associated BioProject, SRA, and Bio-Sample numbers are PRJNA743429, SRR15093617, and SAMN20035122 respectively.
